# Nutritional and metabolic modulation of inflammation in critically ill patients: a narrative review of rationale, evidence and grey areas

**DOI:** 10.1186/s13613-024-01350-x

**Published:** 2024-08-01

**Authors:** Anne-Françoise Rousseau, Robert Martindale

**Affiliations:** 1 Intensive Care Department, University Hospital of Liège, University of Liège, Avenue de l’Hôpital, 1/B35, Liège, B-4000 Belgium; 2 GIGA-I3 Thematic Unit, Inflammation and Enhanced Rehabilitation Laboratory (Intensive Care), GIGA-Research, University of Liège, Liège, Belgium; 3 Department of Surgery, Oregon Health Sciences University, Portland, OR USA

**Keywords:** Inflammation, Critical illness, Intensive care, Burn injury, Nutrition, Metabolism, Stress response, Pharmaconutrition

## Abstract

**Background:**

Inflammation is the hallmark of critical illness and triggers the neuro-endocrine stress response and an oxidative stress. Acute inflammation is initially essential for patient’s survival. However, ongoing or exaggerated inflammation, due to persistent organ dysfunction, immune dysfunction or poor inflammation resolution, is associated to subsequent hypermetabolism and hypercatabolism that severely impact short and long-term functional status, autonomy, as well as health-related costs. Modulation of inflammation is thus tempting, with the goal to improve the short- and long-term outcomes of critically ill patients.

**Findings:**

Inflammation can be modulated by nutritional strategies (including the timing of enteral nutrition initiation, the provision of some specific macronutrients or micronutrients, the use of probiotics) and metabolic treatments. The most interesting strategies seem to be n-3 polyunsaturated fatty acids, vitamin D, antioxidant micronutrients and propranolol, given their safety, their accessibility for clinical use, and their benefits in clinical studies in the specific context of critical care. However, the optimal doses, timing and route of administration are still unknown for most of them. Furthermore, their use in the recovery phase is not well studied and defined.

**Conclusion:**

The rationale to use strategies of inflammation modulation is obvious, based on critical illness pathophysiology and based on the increasingly described effects of some nutritional and pharmacological strategies. Regretfully, there isn’t always substantial proof from clinical research regarding the positive impacts directly brought about by inflammation modulation. Some arguments come from studies performed in severe burn patients, but such results should be transposed to non-burn patients with caution. Further studies are needed to explore how the modulation of inflammation can improve the long-term outcomes after a critical illness.

## Background

A common feature in all critically ill patients is the systemic inflammation that occurs in response to tissue damage (due to infection, trauma or surgery) and proinflammatory mediators. The inflammatory response to tissue damage is induced by a complex array of mediators being interrelated for large part and involves the activation of various cells such as leukocytes and other inflammatory cells, leading to a massive production of reactive oxygen species (ROS). Inflammation initiates a protective response for the destruction of pathogens and serves as chemical attractants for beginning the wound repair. Systemic inflammation further takes the form of a continuum of severity, with potential fluctuations according to the successive hits (for example surgeries, infections). At a cellular level, an impaired mitochondrial function is noted, limiting its metabolic capacities. Inflammation as well triggers a neuro-endocrine stress response, characterized by an adrenergic stimulation, an elevation in cortisol level, a blunted activity of the somatotropic axis and a hypogonadism [1]. In case of ongoing inflammation, the neuroendocrine stress response leads to supraphysiologic metabolic rates (hypermetabolism), protein hypercatabolism (in muscles and bones), insulin resistance, alterations in lipid metabolism and fat composition. Catecholamines are a main effector for hypermetabolism, in link with the browning of white adipose tissue [2]. These metabolic changes are initially essential for patient’s survival, ultimately providing the brain and the immune system with fuel. However, vicious cycles can develop, further potentiating systemic inflammation. Hypercatabolism is associated with immune dysfunction, increasing the susceptibility to recurrent sepsis. Persisting organ injuries, in part due to oxidative stress, trigger recurrent alarmins release: mitochondrial danger-associated molecular patterns (DAMPs) are released from injured or necrotic cells and enhance the inflammatory reaction [3]. As example, mitochondrial oxidative damages have been observed during prolonged mechanical ventilation and diaphragmatic inactivity [4]. The hypothesis of a poor resolution of inflammation, due to defects in pro-resolving pathways, could also participate in chronic inflammation, influenced by age, lifestyle factors, epigenetic or microbiome [5]. A chronic hyperinflammatory hypermetabolic state is a characteristic of chronic critical illness [6]. The definition of what is considered a continuous inflammation and a chronic critical illness is not well defined, is influenced by preadmission clinical conditions and by the severity of the critical illness, but is probably measured in days [7, 8].

A clinically meaningful example of such condition is the severe burn injury (traditionally defined as burns affecting more than 20% of the total body surface area in adults [9]). In these patients, the metabolic derangements are the most intense compared to those encountered in other critical conditions such as sepsis and trauma, and are known to persist for up to years following injury [10]. However, alike observations have been made in non-burn critically ill patients. After COVID-19 acute respiratory distress syndrome (ARDS), a progressive hypermetabolism has been demonstrated during the ICU stay through the use of indirect calorimetry [11]. Signs of persistent inflammation have also been observed 3 months after discharge from intensive care unit (ICU), in association with persistent hypermetabolism and impaired exercise capacity [12, 13]. Similar findings were described in non-COVID ICU survivors [14]. Muscle alterations are attributed partly to atrophy, mitochondrial dysfunction and endoplasmic reticulum stress [15, 16].

The clinical translation of persistent inflammation and subsequent hypermetabolism is significant: functional status, autonomy, return to work or pre-admission level of activities, health-related costs may be severely impacted. These altered outcomes are part of the post-intensive care syndrome [17]. According to a recent systematic review, long-term outcomes following critical illness are potentially associated with an inflammatory process [18]. Trying to modulate persistent inflammation does thus make sense. However, it is wise to recognize that inflammation modulation is still a concept, as the boundary between a beneficial and a detrimental level of systemic inflammation is unknown, and probably specific to a given patient in a given clinical situation, depending on the time point in the illness trajectory. Modifying the inflammation level could aim to reduce the overspill of acute inflammation and/or to prevent further exacerbation and/or to help resolving the response, depending on the considered timing.

Inflammation modulation may be based on nutritional interventions and pharmacological modalities. Nutritional interventions refer to an optimal timing and route of feeding, while pharmaco-nutrition refers to the addition of supraphysiological doses of specific macro- and micronutrients to standard feeding, relying on their ability of acting on inflammatory and immune pathways whatever the nutritional status of the patient [19]. These beneficial effects are dissociated from their nutritional properties, in terms of energy supply for example [20].

The strategies of inflammation modulation that have, according to the current literature, a prominent role while being commercially available in daily practice are presented and discussed in the present narrative review (Table 1). Their potential effects on the subsequent hypermetabolism and hypercatabolism are also described. It should be noted that the presented list may be biased by a subjective selection and omits new or revised drugs that are still being studied in preclinical models [21].

**Table 1:** Recommended, suggested, possible and banned strategies to modulate hyperinflammation in critically ill patients and survivors:

Modulators	Acutely ill patients		Survivors
	Severe burn injury	Medical or surgical critical illness	
Early initiation of nutrition	R as part of the nutritional support [132]	R as part of the nutritional support [38]	
Glutamine	S [37, 132]	S if trauma / chronic wounds [38]	TBE
Arginine	currently NR [132]	currently NR	TBE
Carnitine	S (if deficiency) [102]	S (if deficiency) [102]	TBE
n-3 PUFAs	S (as enriched EN/PN formula) [132]	S (as enriched EN/PN formula) [38]	TBE (as bolus supplement)
Vitamin D	R (as complement or repletion) [132]	R (as complement or repletion) [38, 102]	R (as complement or repletion) [102, 133]
Vitamin C (IV high dose)	OI	OI	
Selenium	R (as complement or repletion) [132]	TBE	TBE (as single high doses)
Probiotics / synbiotics	TBE	TBE	TBE
Propranolol	R [132]	OI	OI
Oxandrolone	R [132]	OI	OI
Insulin intensive therapy	OI	OI	
Metformin	OI	OI	OI
Melatonin	TBE	TBE	TBE

NR: not recommended (signal of harm or absence of benefit); OI: of interest (could be considered in view of the rationale and the available literature, but evidence and practical recommendations still lacking); R: recommended in guidelines; S: suggested in guidelines; TBE: to be explored (real rationale but lack of available literature)

References of available literature and guidelines: refer to the text

Studies in both severe burn patients and non-burn critically ill patients were examined. Severe burn injury is the most severe form of trauma or critical illness in terms of the debilitating stress response it provokes [22], and is thus recognized as a model of systemic inflammation following injury. The hypermetabolic response in severe burn patients has been largely studied, as well as the manipulation of this response, especially because the injury is easy to quantify, as the percentage of body surface area burned is measured objectively. However, exudative losses of proteins and micronutrients make this model particular. The stress response in severe burn patients is also much more intense and prolonged than in trauma or septic patients [23]. Results observed in studies performed in severe burn patients should not be simply transposed to non-burn critically ill patients but are obviously food for thought.

### Clinical assessment of inflammation in critically ill patients

Inflammation monitoring relies on blood biomarkers, mainly acute phase proteins (essentially C-reactive protein (CRP), fibrinogen, serum amyloid A or procalcitonin), cytokines (mainly interleukines (IL) 6 and 10 or TNFα), or immune cells degranulation markers (such as myeloperoxidase). Not all of these markers are readily available in every lab for daily use at bedside.

Conflicting observations mitigate the potential interest of CRP to detect persistent inflammation: CRP remains elevated in burn patients several months after injury [24], while it has been shown to decrease more rapidly than other inflammatory markers in other populations of ICU survivors [12, 13, 25].

Closed monitoring of the inflammation status during the entire ICU and post-ICU trajectory is thus difficult, making individualization of its modulation approximate, if not based on surrogate markers such as body composition or measured energy expenditure.

### Inflammation modulation using medical nutrition

The primary goal of medical nutrition is to provide an adequate supply in nutrients to maintain organ function, to minimize the devastating effects of persistent catabolism and to optimize healing and immune function.

When focusing on inflammation, the timing of medical nutrition initiation could matter, especially when considering enteral route. In severe burn patients, early institution of trophic enteral feeding (i.e. within the first 12 to 24 h after injury) can significantly reduce the level of inflammation, based on plasma C-reactive protein (CRP) levels [26] and can prevent further total energy deficit [27], although the effects of early initiation on hypermetabolism are debated [28]. In non-burn ICU patients, the impact of feeding initiation timing on inflammation markers is still underrecognized, despite the fact that there has been much discussion on the optimal time to provide full nutrition.

The provision of the energy needs after the first days following ICU admission, could also matter. When enable to provide the defined nutritional targets by enteral route, using supplemental parenteral nutrition has been associated with reduced serum levels of pro-inflammatory cytokines and a decrease in CRP over the next 5 days, compared to enteral nutrition alone [29].

Altogether, it can be assumed that early enteral nutrition is a way to preserve gut integrity, subsequently reducing secondary infections, and also reducing toxic bacterial metabolites while enhancing production of short chain fatty acid with local and systemic effects [30]. However, in absence of strong evidence, these effects remain speculative and should be further studied.

Inflammation is known to induce insulin resistance, gastro-intestinal dysfunction and anorexia, essentially limiting the entry of nutrients into cells. In a recent secondary analysis of the EFFORT cohort, including in-hospital patients (outside ICU) at risk of malnutrition, patients with high inflammation (as reflected by CRP levels) tended to benefit less from nutritional support with regards to 30-day mortality, compared to patients with less inflammation [31]. However, it is still unknown if the timing of nutritional support initiation in ICU patients should be individualized according to the inflammation level.

### Inflammation modulation using specific macronutrients

#### Glutamine

Glutamine is the preferred oxidative fuel for rapidly divide cells such as lymphocytes and gut mucosal cells. Glutamine is considered a conditionally essential amino acid: in case of metabolic stress, peripheral demand outstrips the production, leading to depletion. Glutamine is able to attenuate the inflammatory response via effects on heat shock protein, nuclear factor- **κ**B signaling pathway and via the attenuation of tumor necrosis factor- α, IL-6, and IL-18 expression after sepsis [32].

The signal coming from a number of small randomized studies investigating glutamine supplementation in severe burn patients were positive, with a reduction in hospital stay and mortality, as well as gram-negative bacteremia [33]. Another small study also demonstrated that glutamine supplementation could reduce the resting energy expenditure and the catecholamine blood levels in severe burn adults [34]. However, recently, the results of the RE-ENERGIZE multicenter randomized study failed to demonstrate any effect of a 0.5 g/kg/day dose of enteral glutamine on time-to-discharge alive, mortality or gram-negative bacteremia [35]. Importantly glutamine administration was not associated with adverse events. On the contrary, this pragmatic large study included heterogenous patients regarding burn surface area, with very different anticipated exudative glutamine loss [36]. Up to now, in the latest published guidelines on clinical nutrition in ICU, glutamine administration is still suggested in case of major burns [37].

Enteral glutamine administration is also recommended in trauma patients or in patients with complicated wound healing [37, 38]. In other critically ill patients, signal of harm came from the REDOXS study [39], further confirmed by a post-hoc analysis [40], that demonstrated an increased mortality rate in patients with multiple organ failure receiving high doses of intravenous glutamine. The exact mechanism of injury is unknown, possibly related to the accumulation of glutamine or metabolites in patients with renal dysfunction. Considering this alert, as well as negative meta-analysis [41], there is no longer a recommendation for an indiscriminate enteral glutamine supplementation during critical illness [42]. However, an individualized approach with glutamine supplementation in patients with low serum glutamine level or on prolonged parenteral nutrition has never been tested.

#### Arginine

Arginine is required for T lymphocyte functions such as proliferation and the expression of normal T cell receptors. It serves to regulate the appropriate immune response to a catabolic challenge and in vasoregulation via inducible nitric oxide synthase (iNOS), a potent vasodilator. Arginine has been found to improve protein kinetics to wound healing, restore T cell function, and promote the transition from the proinflammatory M1 macrophage to an M2 resolution macrophage [43]. Arginine also appears to support the maturation of myeloid derived suppressor cells (MDSC). MDSCs are a heterogeneous group of immature immune cells that originate in bone marrow that can alter and regulate the immune response to an immune challenge. They are characterized by the increased production of reactive oxygen and nitrogen species, and by increased arginase 1 activity [44]. In excess, MDSCs suppress innate and adaptive immunity and be a major obstacle to a well-orchestrated immune response to challenge. Arginine can enhance the maturation of MDSCs thereby regulating for the appropriate immune response.

L-Arginine supplemented formulations have been in use for greater than four decades with the concept that correction of the relative arginine deficiency noted in critically ill patients would improve systemic immune function. Multiple studies in which supplemental arginine has been delivered in the perioperative period, reported improved clinical outcomes including decreased rates of infections and decreased length of ICU stay [45–47]. Other studies, not all with a strong methodology, also suggested that arginine, supplied enterally at doses available in commercial formulations, may be beneficial to septic patients [48]. In severe burn patients, arginine supplementation has been rarely studied and is not routinely administered as single agent.

Previously a controversy associated with supplemental arginine in critically ill septic patients was discussed. The controversy revolved around the theoretical concept that supplemental arginine would lead to increase nitric oxide via the upregulation of iNOS and result in refractory hypotension. This theory has been now primarily dismissed following multiple human studies. Reports of infusion of arginine in patients with sepsis or septic shock showed no detrimental hemodynamic effects [49, 50].

Altogether, due to weak evidence of clinical benefits, arginine supplementation for all ICU populations is not recommended.

Citrulline is converted to L-arginine through the activity of argininosuccinate synthetase and argininosuccinate lyase. Citrulline has been described as more efficient than L-arginine at increasing plasma arginine [51]. A recent study of enteral citrulline supplementation in mechanically ventilated ICU patients confirmed this description, without demonstrating a subsequent beneficial effect on severity scores or immune biomarkers [52].

#### Carnitine

L-carnitine, a quaternary ammonium, is synthesized endogenously, and for a largest part, ingested via animal-based food. Its main function is the acyl-carnitine carrier transport system of long-chain fatty acids into mitochondria, where they will undergo subsequent β-oxidation.

Carnitine deficiency, defined as a state of tissue or blood carnitine concentration below the requirement for normal organ function, can lead for instance to muscle weakness. Critically ill patients are at risk of carnitine deficiency, due to clinical conditions, supports or treatments, such as prolonged undernutrition, prolonged total parenteral nutrition (commercial formulations do not contain carnitine), prolonged continuous renal replacement therapy (loss of carnitine in the effluent) or valproate treatment (carnitine used for urinary excretion of drug derivates) [53, 54]. According to some rare publications on this topic, carnitine deficiency seems scarce in survivors after ICU discharge, while affecting one third of the survivors 3 months after discharge [55–57].

Clinical cases of carnitine supplementation have been reported in ICU deficient patients during the acute phase [53]. Interventional trials are rare. In a study including patients whatever their carnitine status, a daily 3 g L-carnitine supplementation during ICU stay led to reduced inflammation markers at the end of intervention compared to control group [58]. Same conclusions were drawn in a more recent similar study in septic patients [59]. To date, no study investigated the effects of a carnitine supplementation in ICU survivors. However, there are some indications that L-carnitine supplementation could help reducing some blood biomarkers of inflammation and oxidative stress in various categories of non-critically ill patients [60]. Moreover, growing evidence suggests that L-carnitine could affect muscle mass and function in older adults, probably through a modulation of the muscle protein catabolism / anabolism balance [61].

#### Omega-3 polyunsaturated fatty acids

Omega-3 polyunsaturated fatty acids (n-3 PUFA) found in oily fishes and fish oil supplements have anti-inflammatory effects. The fish oils (EPA and DHA) as well as arachidonic acid are precursors of lipoxins, resolvins, maresins and protectins, these endogenously produced compounds act on several cell lines, including monocytes, macrophages, neutrophils, endothelial and dendritic cells. Collectively these are now referred to as Specialized Proresolving Mediators (SPMs). They can inhibit leucocyte chemotaxis, reduce adhesion between leucocytes and endothelium, decrease the production of eicosanoids from arachidonic acid, and decrease the production of inflammatory cytokines [62]. These effects are translated into improvement of clinical outcomes such as ventilation duration, ICU length of stay and infections when n-3 PUFA are given as parenteral emulsion in parallel with a medical nutrition [63]. Similar positive results have been described with enteral n-3 PUFA [64]: improvement of oxygenation and length of mechanical ventilation in patients with ARDS [65–67], lower mortality in patients with sepsis [68, 69]. SPMs have also been shown to decrease postoperative and neuropathic pain, accelerate removal of inflammatory and necrotic debris, and enhance the conversion from the proinflammatory M1 macrophage to a M2 or resolution macrophage [70].

Independently of their anti-inflammatory effects, n-3 PUFAs have anabolic effect in muscles, at least partially mediated via the activation of the mTOR pathway [71]. A recent meta-analysis of human studies in heterogeneous population groups identified that lean mass and skeletal muscle mass are favored by higher intakes of EPA and DHA [72]. Finally, another interesting metabolic perspective comes from an old study in severe burn adults: when receiving a low-fat diet enriched with n-3 PUFA, patients had significantly higher insulin-like growth factor-1 (IGF-1) one month after injury compared to patients receiving high-fat diet or low-fat diet without n-3 PUFA [73]. However, the functional consequence of such endocrine modulation is still to be determined.

Altogether, the use of n-3 PUFAs during the critical care trajectory is relevant and attractive, but future research needs to focus on the best dosages of omega-3 PUFAs to use, the optimal route(s) of administration, the optimal timing and which patients are most likely to benefit [74].

### Inflammation modulation using micronutrients

#### Micronutrients with direct anti-inflammatory effects

Vitamin D has a wide range of action, including the classical bone mineralization and remodeling [75]. Vitamin D has also anti-inflammatory properties, inhibiting the activation of nuclear factor-kappa B (NF-κB) and the production of pro-inflammatory cytokines, such as IL-6 and TNF-α [76]. Some human data suggest that vitamin D could regulate mitochondrial function, especially in muscle, with potential benefits on muscle strength or recovery after exercises [77]. Translation of these findings in the critical care context is currently lacking. From a general point of view, extra-skeletal effects of vitamin D remain debated, mainly due to methodological concerns in the available literature [78].

In non-critically ill patients, vitamin D supplementation had significant effects on inflammation and oxidative stress [79, 80]. Such effects have been observed in a small study including deficient patients with sepsis who received high dose boluses of cholecalciferol [81]. However, to date, it is still needed to verify whether the effects of vitamin D on inflammation and mitochondria, through a repletion or a supplementation strategy, modify the short and long-term outcomes of ICU patients or survivors.

Unfortunately, the optimal vitamin D prescription in the critical care context is undetermined, whether in terms of dose, route or timing.

Due to a very high prevalence of hypovitaminosis D in critically ill patients [82] (and even more in severe burn patients [83]),it is largely recommended to complete or replete in vitamin D3 to reach a normal vitamin D status [84]. The oral or enteral route is usually used for this purpose. However, considering the gut oedema and the multiple organ dysfunction observed in critically ill patients, it is unclear if the inactive cholecalciferol is adequately absorbed by the gastro-intestinal tract and further converted in its biological active form. These physiological derangements could be the rationale for a more pronounced effect of vitamin D administration on overall mortality or ICU length of stay when given by parenteral route [85]. Patients presenting a severe vitamin D deficiency could be those who would benefit the most from a vitamin D repletion or supplementation [86, 87]. It is now increasingly recognized from studies in other medical conditions that ultra-high bolus doses of vitamin D (> 100,000 IU) is ineffective due to the subsequent long term activation of the inactivating 24-hydroxylase and fibroblast growth factor 23 [88].

#### Micronutrients with antioxidant effects

Oxidative stress is defined as a state in which the level of toxic ROS overcome the endogenous antioxidant defenses of the host, and damage biologically relevant molecules, leading to mitochondrial damages and systemic inflammation acceleration. Copper, manganese, zinc, iron and selenium are required for the enzymatic endogenous antioxidant defense, while the non-enzymatic mechanisms include vitamins A, C, E [89].

Exogenous antioxidants have been considered for years as part of the ICU nutritional management in attempt to limit the oxidative stress associated critical illness and to improve outcomes such as mortality or illness severity [90, 91]. Despite active research, study results are often conflicting. The variability of the inflammatory response, the oxidative stress, and the endogenous immune response combined with the heterogeneity of the ICU population (in terms of critical condition and amount of exogenous antioxidants required to restore the antioxidant capacity) makes the dosing judgement of antioxidants unclear. The issue of timing of antioxidant administration is also a key factor, as well as the ideal combination of antioxidants [92]. These are challenging questions to respond to, particularly given that laboratory tests of micronutrient status are an area prone to misuse and misinterpretation [93].

Vitamin C has been extensively studied in different critical care settings over the past years. In severe burn injury, it has been demonstrated more than 30 years ago that high intravenous doses of vitamin C in the early phase following injury could reduce fluid requirements and burn edema [94, 95]. The ongoing VICToRY Trial aims to bring a higher level of evidence regarding this sparing strategy (ClinicalTrials.gov Identifier: NCT04138394). According to two recent meta-analysis in non burn critically ill patients, high dose intravenous vitamin C monotherapy could be associated with a trend toward reduction in overall mortality [96, 97]. Interestingly, a high-dose antioxidant supplementation protocol including vitamin C, administered to surgical and trauma patients, led to a reduction of the inflammatory response [98]. Used alone, in a small cohort of patients with severe sepsis, intravenous high dose of vitamin C significantly reduced inflammatory biomarkers [99]. The safety of high doses of vitamin C is still questioned, especially regarding nephropathy and hemolytic anemia in ignored cases of Glucose-6-Phosphate Dehydrogenase deficiency: data providing from published studies are conflicting [100].

Some weak trends of an inflammation modulation by selenium supplementation in monotherapy has been observed in small cohorts of critically patients at high risk of bias [101]. Dose and timing of selenium supplementation is unclear, especially since selenium has pro-oxidant properties which can lead to the opposite of the desired effect.

The use of other antioxidant micronutrients, with the aim at modulating inflammation during or after ICU stay, has unfortunately not been widely studied in clinical practice.

Overall, at the present time, the guidelines maintain the use of repletion dose of antioxidant micronutrients, and not the use of very high doses [102].

### Inflammation modulation using probiotics

Dysbiosis is the disruption of the homeostasis of the microbiota. In ICU patients, intestinal dysbiosis is driven by numerous factors including antimicrobial treatment, the change in nutritional pattern, the splanchnic hypoperfusion and the stress of the acute illness [103]. Emergence of a pathobiome (reduced diversity and changes to a more pathogenic flora) is observed within hours following varied insults such as trauma, sepsis or extended burns [104]. Alterations in intestinal homeostasis and gut microbiota in critical illness have been associated with increased inflammatory cytokine production [105], possibly due to the passage of microbial products in the circulation via the mesenteric lymph. The repletion of the gut with health-promoting probiotics (living microbes of human origin), potentially combined with prebiotics (synbiotics), has been hypothesized as a promising strategy to maintain the intestinal homeostasis. A recent meta-analysis including 33 studies examined the efficacy of probiotics or synbiotics in critically ill patients and concluded they could significantly reduce the incidence of ventilation associated pneumonia, and decrease the ICU length of stay and mortality as well [106].

The impact of probiotics or symbiotics administration on the pro-inflammatory/anti-inflammatory balance in critically ill patients is unclear [107]. Small studies showed interesting effects of synbiotics on different inflammation markers in various contexts such as unselected acute disease [108], severe acute pancreatitis [109] or hepatectomy [110].

Importantly, the studies about microbiota-targeted therapies suffer from a huge heterogeneity in both patients and compounds tested. Therefore, drawing firm conclusions remains hazardous [111]. Moreover, concerns regarding the safety of probiotics exist, especially in the context of acute critical illness [112]. Due to their immunocompromised status, critically ill patients are at risk of bacteremia or fungemia secondary to probiotics administration [113].

In the future, microbiome modulation may become a novel adjuvant strategy in inflammation modulation [114]. A final aim could be to prevent or even treat ICU-related sarcopenia and progression to chronic critical illness. There is promising research underway that should contribute to the understanding of how to use individualised microbiota-targeted treatment [115].

### Inflammation modulation using metabolic treatments

The blockade of β-adrenergic stimulation with propranolol is used to blunt the catecholamine-mediated hypermetabolism. This effect has been confirmed in studies performed in burn patients during the acute phase as well as during rehabilitation [116, 117]. In severe burn children, propranolol given after 7 days following injury significantly decreased TNF-α and IL-1b. According to a recent metabolomic analysis, propranolol appears to mitigate some of the pathophysiologic effects of stress including lipidic metabolism, mitochondrial function and endoplasmic reticulum stress [118]. Evidence of benefits in non-burn ICU patients is currently lacking.

Oxandrolone, an oral synthetic testosterone analog, has been used for decades in acute and rehabilitating severe burn patients with beneficial effects on body composition and wound healing, while being safe for liver function [119]. More recently, its effect on inflammatory response was investigated in a murine model of burn injury. Interestingly, oxandrolone treatment during the first days after injury led to a generalized reduction of pro-inflammatory mediators and an oxidative stress suppression in some organs [120]. Up to now, oxandrolone has never been studied in chronic critical illness or in ICU survivors. Unfortunately, oxandrolone is not available in some countries, but could then be substituted by nandrolone decanoate [121].

Insulin at high dose is considered as an anabolic agent, by increasing muscle protein turnover. It is thought to act by increasing bioavailability of IGF1, and also by anti-inflammatory properties. Indeed, in severe burn patients, insulin treatment decreased expression of proinflammatory cytokines, increased anti-inflammatory cytokines, and modulated the synthesis of acute phase proteins [122]. Same findings were detected in non-burn critically ill patients with prolonged stay in ICU [123] In similar patients, intensive insulin therapy was also found to be associated with a decrease in cortisol blood level, independently of any effect on cortisol binding protein [124].However, the effective dose (around 0.0015 U/kg/min) is greater than the one used to maintain euglycemia (glycemia < 180 mg/dL) during acute critical illness, and exposes to a risk of hypoglycemia that can be mitigated by administration of exogenous glucose.

Metformin is an antihyperglycemic agent acting by decreasing hepatic glucose production while increasing muscle glucose uptake. Metformin has anti-inflammatory properties through inhibition of the NF-κB activation [125]. In patients with diabetes mellitus admitted in ICU for sepsis, preadmission metformin use may reduce mortality [126]. The underlying mechanism is unclear, but probably multifactorial in relation with the different properties of metformin: mitigation of inflammation, autophagy and mitochondrial functions [127]. In a model of mice suffering from sepsis, metformin treatment decreased the messenger ribonucleic acid levels of inflammatory factors and cytokines [128]. Metformin has also been demonstrated to have anabolic properties in severe burn patients, probably as a consequence of glycemia control. Promising data were recently published in a mice model of thermal injury of moderate severity, showing a preservation of muscle mass and a proliferation of muscle satellite cells in treated animals [129]. The treatment is rarely associated with hypoglycemia and is now widely studied in non-diabetics [130]. However, its properties other than anti-hyperglycemic have never been studied in ICU survivors.

Melatonin, a pleiotropic hormone secreted by the pineal gland, plays roles in circadian rhythm, immune regulation and energy metabolism. In recent years, its administration as dietary supplement has gain popularity, especially for its protective role in sarcopenia related diseases. Indeed, among its protective effects on ICU-acquired weakness through reduction of proteolysis and improvement of mitochondrial dysfunction, melatonin has been shown, mainly in animal models, to have a direct anti-inflammatory effect [131]. Data on its use during acute illness or during rehabilitation aiming at enhancing muscle recovery are lacking, although this treatment seems to be safe at doses usually used for sleep promotion.

## Conclusion

Numerous strategies of inflammation attenuation, based on the physiologic effects of nutrients or pharmacological agents, are available in human clinical practice. Regretfully, there isn’t always substantial proof from clinical research regarding the positive impacts that are directly brought about by inflammation modulation. Some arguments come from small studies performed in severe burn patients, but such results should be transposed to non-burn patients with caution. Given the metabolic effects of persisting inflammation and their long-term consequences, it makes sense to modulate it. However, the ideal timing for inflammation modulation is still undetermined, as well as the doses or the potential synergic effects of the different strategies. The ultimate goal is to individualize the modulation of inflammation, but this is made difficult by the imprecise or ambiguous clinical monitoring of inflammation that is available at the bedside. In the next future, further studies are needed to explore how the modulation of inflammation can improve the long-term outcomes after a critical illness.

## Data Availability

Not applicable.

## Abbreviations

ARDS: acute respiratory distress syndrome
BCAA: branched chain amino acid
CRP: C-reactive protein
EPA: eicosapentaenoic acid
DHA: docosahexaenoic acid
HMB: ß-hydroxy-b-methylbutyrate
ICU: intensive care unit
IGF-1: insulin-like growth factor-1
IGFBP-3: IGF binding protein-3
MDSC: myeloid derived suppressor cell
n-3 PUFA: omega-3 polyunsaturated fatty acids
rhGH: recombinant human growth hormone
SPM: specialized proresolving mediator
SPN: supplemental parenteral nutrition
